# (2*R*)-2-Cinnamoylamino-*N*-[5-(4-methoxy­phen­yl)-1,3,4-thia­diazol-2-yl]propanamide

**DOI:** 10.1107/S1600536808027803

**Published:** 2008-09-06

**Authors:** Shao-Hua Li, Gang Li, Hui-Ming Huang, Guo-Gang Tu, Cheng-Mei Liu

**Affiliations:** a State Key Laboratory of Food Science and Technology, Nanchang University, 330047 Nanchang, JiangXi, People’s Republic of China; b Department of Pharmacy, NanChang University Medical College, 330006 Nanchang, JiangXi, People’s Republic of China

## Abstract

The asymmetric unit of the title compound, C_21_H_20_N_4_O_3_S, contains two independent mol­ecules. The dihedral angles between the two benzene rings are 47.6 (1) and 30.2 (1)°, the corresponding values between the *p*-methoxy­benzene and thia­diazol rings are 12.3 (1) and 24.7 (1)°, respectively, for the two mol­ecules. The conformations of the N—H and C=O bonds are *anti* with respect to each other. The enone groups show a *trans* configuration. The crystal structure is stabilized by N—H⋯O and N—H⋯N inter­actions. The absolute structure could not be determined from the X-ray data but the absolute configuration has been assigned by reference to an unchanging chiral centre in the synthetic procedure.

## Related literature

For 1,3,4-thiadiazole scaffold compounds and their biological activity, see: Tu *et al.* (2008 ▶). For the synthesis, see: Foroumadi *et al.* (1999 ▶); Levy & Palmer (1942 ▶); Song *et al.* (1992 ▶). For related structures, see: Fun *et al.* (2008 ▶); Gowda *et al.* (2008 ▶) Thiruvalluvar *et al.* (2008 ▶).
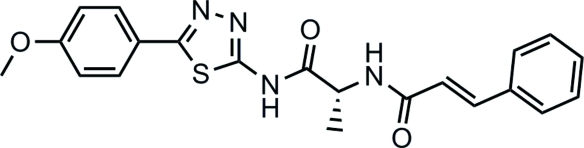


## Experimental

### 

#### Crystal data

C_21_H_20_N_4_O_3_S
*M* *_r_* = 408.48
Triclinic, 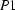

*a* = 9.082 (3) Å
*b* = 9.849 (3) Å
*c* = 13.644 (4) Å
α = 79.587 (4)°
β = 83.253 (4)°
γ = 65.458 (4)°
*V* = 1090.8 (5) Å^3^
*Z* = 2
Mo *K*α radiation
μ = 0.18 mm^−1^
*T* = 296 (2) K
0.35 × 0.24 × 0.06 mm

#### Data collection

Bruker APEXII CCD area-detector diffractometer
Absorption correction: none
6219 measured reflections
3553 independent reflections
2735 reflections with *I* > 2σ(*I*)
*R*_int_ = 0.022

#### Refinement

*R*[*F* ^2^ > 2σ(*F* ^2^)] = 0.038
*wR*(*F* ^2^) = 0.097
*S* = 0.94
3553 reflections
528 parameters
3 restraints
H-atom parameters constrained
Δρ_max_ = 0.20 e Å^−3^
Δρ_min_ = −0.17 e Å^−3^

### 

Data collection: *APEX2* (Bruker, 2004 ▶); cell refinement: *APEX2* and *SAINT* (Bruker, 2004 ▶); data reduction: *SAINT*; program(s) used to solve structure: *SHELXS97* (Sheldrick, 2008 ▶); program(s) used to refine structure: *SHELXL97* (Sheldrick, 2008 ▶); molecular graphics: *APEX2*; software used to prepare material for publication: *APEX2* and *publCIF* (Westrip, 2008 ▶).

## Supplementary Material

Crystal structure: contains datablocks I. DOI: 10.1107/S1600536808027803/im2079sup1.cif

Structure factors: contains datablocks I. DOI: 10.1107/S1600536808027803/im2079Isup2.hkl

Additional supplementary materials:  crystallographic information; 3D view; checkCIF report

## Figures and Tables

**Table 1:** Hydrogen-bond geometry (Å, °)

*D*—H⋯*A*	*D*—H	H⋯*A*	*D*⋯*A*	*D*—H⋯*A*
N3—H3*A*⋯O4	0.86	2.10	2.960 (4)	177
N4—H4*A*⋯N6^i^	0.86	2.17	3.016 (5)	168
N7—H7*A*⋯O1^ii^	0.86	2.09	2.944 (4)	171
N8—H8⋯N2	0.86	2.20	3.036 (5)	165

Symmetry codes: (i) 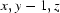; (ii) 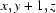.

**Fig. 1.**
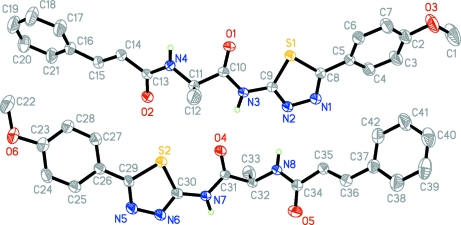
Molecular structure of the two independent molecules of the title compound. Displacement ellipsoids are drawn at the 50% probability level.

**Fig. 2.**
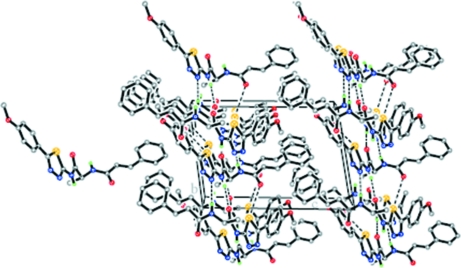
The crystal packing of title compound, viewed along the a axis with hydrogen bonds drawn as dashed lines.

## supplementary crystallographic information

### Comment

In our previous work, 1,3,4-thiadiazole scaffold compounds and their biological activity have been studied (Tu *et al.*, 2008). In view of the importance of these organic materials, the title compound (Fig. 1) was synthesized (Foroumadi *et al.*, 1999; Levy & Palmer 1942; Song *et al.*, 1992) and its crystal structure is reported here. The asymmetric unit of the title compound, C_21_H_20_N_4_O_3_S, contains two independent molecules. The dihedral angles between the *p*-methoxybenzene and thiadiazol rings is 12.2 (1) ° and 24.7 (1)°, the corresponding values between the two benzene rings are measured to 47.6 (1)° and 30.2 (1)°, respectively, for the two molecules. The conformations of the N—H and C=O bonds are anti with respect to each other. The enone groups are *trans* configurated. Bond lengths and angles are in normal ranges and comparable to those in related structures (Gowda *et al.*, 2008; Fun *et al.*, 2008; Thiruvalluvar *et al.*, 2008). In the crystal structure, molecules are linked through intermolecular N—H···O hydrogen bonds forming a three-dimensional network (Table 1, Figure 2).

### Experimental

*N*,*N*-Dicyclohexylcarbodiimide (5.7 mmol) was added to a cooled solution of N-cinnamoyl-D-alanine (5.6 mmol) and *N*-hydroxysuccinimide (5.6 mmol) in freshly distilled dioxane (30 ml). The reaction mixture was stirred overnight at room temperature. The insoluble material was filtered off and washed with cold dioxane. 2-Amino-5-(4-methoxyphenyl)-1,3,4-thiadiazole (5.5 mmol) was added to the filtrate and the reaction mixture was stirred for 48 hr at room temperature. The solvent was removed under reduced pressure. The residual was dissolved in EtOAc and the insoluble material was filtered off. The filtrate was washed successively with saturated Na_2_CO_3_ solution (20 ml, *x* 3), water (20 ml, *x* 1), 0.1 *M* HCl (20 ml, *x* 3) and water (20 ml, *x* 1). The organic layer was evaporated *in vacuo* and the residue was recrystallized from methanol (30 ml), yield: 35.2%. Colorless block-shaped single crystals of the title compound suitable for X-ray diffraction analysis precipitated after several days.

### Refinement

H atoms were positioned geometrically and refined using a riding model using *SHELXL97* default values (*U*_ĩso_(H) = 1.2 *U*_eq_(C) for CH and CH_2_ groups and U_ĩso_(H) = 1.5 U_eq_(C) for CH_3_). Refinement with all data (Friedel opposites not merged) led to an unsuitably large error of the Flack parameter. The final refinement was therefore performed with a data set with merged Friedel pairs, hence the calculated Flack parameter is meaningless. The absolute configuration is nevertheless undoubtly as described since enantiomerically pure starting compounds were used and the reaction conditions are not condidered to lead to racemisation or inversion.

### Crystal data

**Table:**

C_21_H_20_N_4_O_3_S	*Z* = 2
*M**_r_* = 408.48	*F*(000) = 428
Triclinic, *P*1	*D*_x_ = 1.244 Mg m^−^^3^
Hall symbol: P 1	Melting point: 450 K
*a* = 9.082 (3) Å	Mo *K*α radiation, λ = 0.71073 Å
*b* = 9.849 (3) Å	Cell parameters from 2616 reflections
*c* = 13.644 (4) Å	θ = 2.3–25.0°
α = 79.587 (4)°	µ = 0.18 mm^−^^1^
β = 83.253 (4)°	*T* = 296 K
γ = 65.458 (4)°	Bolck, colourles
*V* = 1090.8 (5) Å^3^	0.35 × 0.25 × 0.06 mm

### Data collection

**Table:**

Bruker APEXII CCD area-detector diffractometer	2735 reflections with *I* > 2σ(*I*)
Radiation source: fine-focus sealed tube	*R*_int_ = 0.022
graphite	θ_max_ = 25.0°, θ_min_ = 2.3°
phi and ω scans	*h* = −10→10
6219 measured reflections	*k* = −11→11
3553 independent reflections	*l* = −16→16

### Refinement

**Table:**

Refinement on *F*^2^	Primary atom site location: structure-invariant direct methods
Least-squares matrix: full	Secondary atom site location: difference Fourier map
*R*[*F*^2^ > 2σ(*F*^2^)] = 0.038	Hydrogen site location: inferred from neighbouring sites
*wR*(*F*^2^) = 0.097	H-atom parameters constrained
*S* = 0.94	*w* = 1/[σ^2^(*F*_o_^2^) + (0.0648*P*)^2^] where *P* = (*F*_o_^2^ + 2*F*_c_^2^)/3
3553 reflections	(Δ/σ)_max_ < 0.001
528 parameters	Δρ_max_ = 0.20 e Å^−^^3^
3 restraints	Δρ_min_ = −0.18 e Å^−^^3^

### Special details

**Table:**

Geometry. All e.s.d.'s (except the e.s.d. in the dihedral angle between two l.s. planes) are estimated using the full covariance matrix. The cell e.s.d.'s are taken into account individually in the estimation of e.s.d.'s in distances, angles and torsion angles; correlations between e.s.d.'s in cell parameters are only used when they are defined by crystal symmetry. An approximate (isotropic) treatment of cell e.s.d.'s is used for estimating e.s.d.'s involving l.s. planes.
Refinement. Refinement of *F*^2^ against ALL reflections. The weighted *R*-factor *wR* and goodness of fit *S* are based on *F*^2^, conventional *R*-factors *R* are based on *F*, with *F* set to zero for negative *F*^2^. The threshold expression of *F*^2^ > σ(*F*^2^) is used only for calculating *R*-factors(gt) *etc*. and is not relevant to the choice of reflections for refinement. *R*-factors based on *F*^2^ are statistically about twice as large as those based on *F*, and *R*- factors based on ALL data will be even larger.# SQUEEZE RESULTS (APPEND TO CIF) loop_ _platon_squeeze_void_nr _platon_squeeze_void_average_x _platon_squeeze_void_average_y _platon_squeeze_void_average_z _platon_squeeze_void_volume _platon_squeeze_void_count_electrons 1 0.151 0.591 0.943 19.4 0.7 2 0.269 -0.200 0.408 64.4 11.5

### Fractional atomic coordinates and isotropic or equivalent isotropic displacement parameters (Å^2^)

**Table:**

	*x*	*y*	*z*	*U*_iso_*/*U*_eq_	
C1	−1.0057 (8)	0.2529 (9)	0.5939 (8)	0.166 (4)	
H1A	−0.9914	0.3381	0.6089	0.249*	
H1B	−1.0630	0.2174	0.6489	0.249*	
H1C	−1.0669	0.2824	0.5351	0.249*	
C2	−0.7380 (5)	0.1750 (6)	0.5178 (4)	0.0752 (13)	
C3	−0.7671 (6)	0.3158 (6)	0.4658 (4)	0.0792 (15)	
H3	−0.8694	0.3938	0.4689	0.095*	
C4	−0.6419 (5)	0.3393 (5)	0.4090 (4)	0.0699 (12)	
H4	−0.6612	0.4347	0.3736	0.084*	
C5	−0.4885 (5)	0.2259 (5)	0.4028 (3)	0.0527 (10)	
C6	−0.4629 (5)	0.0841 (5)	0.4548 (3)	0.0623 (11)	
H6	−0.3611	0.0056	0.4508	0.075*	
C7	−0.5874 (6)	0.0576 (6)	0.5131 (4)	0.0764 (13)	
H7	−0.5694	−0.0377	0.5483	0.092*	
C8	−0.3565 (5)	0.2565 (5)	0.3439 (3)	0.0534 (10)	
C9	−0.1214 (5)	0.2590 (5)	0.2508 (3)	0.0532 (10)	
C10	0.1343 (5)	0.1053 (4)	0.1705 (3)	0.0510 (10)	
C11	0.2964 (5)	0.1100 (5)	0.1366 (3)	0.0565 (10)	
H11	0.2780	0.2140	0.1076	0.068*	
C12	0.3982 (6)	0.0670 (7)	0.2263 (4)	0.0879 (16)	
H12A	0.4259	−0.0374	0.2523	0.132*	
H12B	0.3379	0.1281	0.2767	0.132*	
H12C	0.4954	0.0831	0.2068	0.132*	
C13	0.4459 (4)	0.0663 (4)	−0.0225 (3)	0.0483 (9)	
C14	0.5593 (5)	−0.0498 (5)	−0.0819 (3)	0.0544 (10)	
H14	0.5798	−0.1511	−0.0612	0.065*	
C15	0.6316 (5)	−0.0082 (5)	−0.1650 (3)	0.0589 (10)	
H15	0.5954	0.0950	−0.1866	0.071*	
C16	0.7605 (5)	−0.1055 (5)	−0.2260 (3)	0.0609 (11)	
C17	0.8502 (6)	−0.2567 (6)	−0.1942 (4)	0.0853 (15)	
H17	0.8253	−0.3019	−0.1323	0.102*	
C18	0.9750 (7)	−0.3400 (7)	−0.2534 (5)	0.106 (2)	
H18	1.0335	−0.4416	−0.2305	0.128*	
C19	1.0168 (7)	−0.2809 (8)	−0.3434 (5)	0.0972 (18)	
H19	1.1035	−0.3401	−0.3816	0.117*	
C20	0.9305 (7)	−0.1341 (8)	−0.3774 (4)	0.0917 (17)	
H20	0.9566	−0.0923	−0.4401	0.110*	
C21	0.8028 (6)	−0.0446 (6)	−0.3194 (3)	0.0773 (14)	
H21	0.7454	0.0567	−0.3433	0.093*	
C22	1.1903 (8)	−0.0712 (8)	−0.1755 (6)	0.115 (2)	
H22A	1.1687	−0.1288	−0.1153	0.173*	
H22B	1.2980	−0.1256	−0.2015	0.173*	
H22C	1.1135	−0.0544	−0.2238	0.173*	
C23	1.0387 (5)	0.1557 (6)	−0.1050 (4)	0.0679 (12)	
C24	1.0413 (5)	0.2832 (6)	−0.0771 (4)	0.0708 (13)	
H24	1.1329	0.3043	−0.0914	0.085*	
C25	0.9095 (5)	0.3767 (5)	−0.0289 (4)	0.0643 (11)	
H25	0.9130	0.4609	−0.0092	0.077*	
C26	0.7698 (5)	0.3508 (5)	−0.0081 (3)	0.0543 (10)	
C27	0.7702 (5)	0.2255 (5)	−0.0358 (4)	0.0681 (12)	
H27	0.6773	0.2062	−0.0227	0.082*	
C28	0.9036 (6)	0.1251 (6)	−0.0830 (4)	0.0763 (13)	
H28	0.9016	0.0383	−0.0994	0.092*	
C29	0.6258 (5)	0.4600 (4)	0.0389 (3)	0.0537 (10)	
C30	0.3751 (5)	0.6067 (5)	0.1151 (3)	0.0549 (10)	
C31	0.1097 (5)	0.6158 (4)	0.1795 (3)	0.0488 (9)	
C32	−0.0580 (5)	0.7268 (4)	0.2076 (3)	0.0519 (10)	
H32	−0.0476	0.8095	0.2325	0.062*	
C33	−0.1544 (6)	0.7907 (6)	0.1143 (4)	0.0846 (15)	
H33A	−0.1650	0.7107	0.0887	0.127*	
H33B	−0.0993	0.8378	0.0648	0.127*	
H33C	−0.2601	0.8641	0.1301	0.127*	
C34	−0.1692 (5)	0.6921 (5)	0.3768 (3)	0.0572 (10)	
C35	−0.2660 (5)	0.6200 (5)	0.4430 (3)	0.0636 (11)	
H35	−0.3026	0.5591	0.4171	0.076*	
C36	−0.3019 (5)	0.6389 (5)	0.5363 (3)	0.0638 (11)	
H36	−0.2649	0.7022	0.5589	0.077*	
C37	−0.3926 (5)	0.5732 (5)	0.6091 (3)	0.0626 (11)	
C38	−0.4185 (7)	0.6053 (7)	0.7051 (4)	0.0899 (16)	
H38	−0.3807	0.6716	0.7228	0.108*	
C39	−0.5020 (8)	0.5383 (9)	0.7766 (4)	0.105 (2)	
H39	−0.5189	0.5604	0.8415	0.126*	
C40	−0.5591 (7)	0.4401 (7)	0.7516 (5)	0.0953 (18)	
H40	−0.6158	0.3970	0.7988	0.114*	
C41	−0.5316 (8)	0.4077 (7)	0.6580 (5)	0.0948 (17)	
H41	−0.5690	0.3408	0.6408	0.114*	
C42	−0.4493 (6)	0.4714 (6)	0.5874 (4)	0.0807 (14)	
H42	−0.4310	0.4459	0.5233	0.097*	
N1	−0.3581 (4)	0.3920 (4)	0.3237 (3)	0.0643 (9)	
N2	−0.2200 (4)	0.3929 (4)	0.2696 (3)	0.0641 (9)	
N3	0.0283 (4)	0.2369 (4)	0.2044 (2)	0.0555 (8)	
H3A	0.0578	0.3109	0.1960	0.067*	
N4	0.3805 (4)	0.0138 (4)	0.0618 (2)	0.0556 (8)	
H4A	0.3890	−0.0778	0.0710	0.067*	
N5	0.6130 (4)	0.5942 (4)	0.0479 (3)	0.0669 (10)	
N6	0.4668 (4)	0.6790 (4)	0.0918 (3)	0.0668 (10)	
N7	0.2200 (4)	0.6771 (4)	0.1557 (3)	0.0567 (8)	
H7A	0.1911	0.7681	0.1667	0.068*	
N8	−0.1393 (4)	0.6583 (4)	0.2839 (3)	0.0603 (9)	
H8	−0.1699	0.5930	0.2693	0.072*	
O1	0.1002 (3)	−0.0005 (3)	0.1732 (2)	0.0627 (8)	
O2	0.4184 (3)	0.2007 (3)	−0.0448 (2)	0.0634 (7)	
O4	0.1420 (3)	0.4837 (3)	0.1726 (2)	0.0652 (8)	
O5	−0.1232 (5)	0.7795 (4)	0.4039 (2)	0.0849 (10)	
O3	−0.8515 (5)	0.1357 (5)	0.5771 (4)	0.1156 (15)	
O6	1.1766 (4)	0.0707 (4)	−0.1543 (3)	0.0913 (11)	
S1	−0.18753 (12)	0.11853 (11)	0.29467 (8)	0.0607 (3)	
S2	0.45630 (12)	0.42627 (11)	0.08513 (8)	0.0581 (3)	

### Atomic displacement parameters (Å^2^)

**Table:**

	*U*^11^	*U*^22^	*U*^33^	*U*^12^	*U*^13^	*U*^23^
C1	0.080 (4)	0.109 (6)	0.259 (10)	−0.022 (4)	0.080 (6)	0.002 (6)
C2	0.050 (3)	0.069 (3)	0.102 (3)	−0.025 (2)	0.016 (2)	−0.012 (3)
C3	0.051 (3)	0.057 (3)	0.119 (4)	−0.020 (2)	0.010 (3)	−0.001 (3)
C4	0.062 (3)	0.054 (3)	0.090 (3)	−0.023 (2)	0.012 (2)	−0.009 (2)
C5	0.051 (2)	0.052 (2)	0.062 (2)	−0.027 (2)	0.0095 (18)	−0.0178 (19)
C6	0.053 (2)	0.052 (2)	0.076 (3)	−0.018 (2)	0.011 (2)	−0.013 (2)
C7	0.065 (3)	0.064 (3)	0.093 (3)	−0.028 (2)	0.014 (2)	−0.001 (2)
C8	0.056 (2)	0.057 (3)	0.058 (2)	−0.033 (2)	0.0139 (18)	−0.0210 (19)
C9	0.060 (2)	0.052 (2)	0.059 (2)	−0.033 (2)	0.0153 (18)	−0.0218 (18)
C10	0.060 (2)	0.042 (2)	0.057 (2)	−0.029 (2)	0.0127 (19)	−0.0136 (18)
C11	0.063 (2)	0.049 (2)	0.068 (3)	−0.033 (2)	0.019 (2)	−0.023 (2)
C12	0.074 (3)	0.113 (5)	0.086 (4)	−0.040 (3)	0.009 (3)	−0.039 (3)
C13	0.048 (2)	0.047 (2)	0.056 (2)	−0.0267 (19)	0.0089 (17)	−0.0127 (18)
C14	0.049 (2)	0.052 (2)	0.065 (3)	−0.0241 (19)	0.0119 (19)	−0.0139 (19)
C15	0.054 (2)	0.059 (3)	0.067 (3)	−0.027 (2)	0.0130 (19)	−0.015 (2)
C16	0.058 (2)	0.067 (3)	0.060 (2)	−0.027 (2)	0.0108 (19)	−0.020 (2)
C17	0.082 (3)	0.079 (4)	0.075 (3)	−0.020 (3)	0.022 (3)	−0.011 (3)
C18	0.091 (4)	0.089 (4)	0.104 (4)	−0.010 (3)	0.036 (3)	−0.020 (3)
C19	0.084 (4)	0.097 (5)	0.096 (4)	−0.022 (3)	0.030 (3)	−0.036 (3)
C20	0.094 (4)	0.114 (5)	0.067 (3)	−0.045 (4)	0.033 (3)	−0.029 (3)
C21	0.071 (3)	0.082 (3)	0.070 (3)	−0.028 (3)	0.023 (2)	−0.012 (2)
C22	0.077 (4)	0.108 (5)	0.150 (6)	−0.013 (3)	0.020 (4)	−0.066 (4)
C23	0.053 (2)	0.066 (3)	0.077 (3)	−0.015 (2)	0.006 (2)	−0.018 (2)
C24	0.050 (3)	0.067 (3)	0.096 (3)	−0.029 (2)	0.013 (2)	−0.010 (3)
C25	0.053 (2)	0.050 (2)	0.095 (3)	−0.025 (2)	0.007 (2)	−0.018 (2)
C26	0.046 (2)	0.048 (2)	0.070 (3)	−0.0207 (19)	0.0022 (19)	−0.011 (2)
C27	0.051 (2)	0.060 (3)	0.096 (3)	−0.024 (2)	0.009 (2)	−0.021 (2)
C28	0.058 (3)	0.072 (3)	0.105 (4)	−0.024 (2)	0.012 (2)	−0.042 (3)
C29	0.047 (2)	0.047 (2)	0.069 (2)	−0.0249 (19)	0.0118 (18)	−0.0095 (19)
C30	0.057 (2)	0.043 (2)	0.069 (2)	−0.026 (2)	0.0072 (19)	−0.0100 (18)
C31	0.058 (2)	0.039 (2)	0.057 (2)	−0.0277 (19)	0.0127 (18)	−0.0125 (17)
C32	0.050 (2)	0.038 (2)	0.074 (3)	−0.0247 (18)	0.0155 (19)	−0.0167 (18)
C33	0.066 (3)	0.076 (3)	0.099 (4)	−0.024 (3)	0.005 (3)	0.001 (3)
C34	0.059 (2)	0.048 (2)	0.069 (3)	−0.028 (2)	0.014 (2)	−0.016 (2)
C35	0.064 (2)	0.057 (3)	0.079 (3)	−0.034 (2)	0.023 (2)	−0.023 (2)
C36	0.069 (3)	0.060 (3)	0.068 (3)	−0.033 (2)	0.011 (2)	−0.015 (2)
C37	0.067 (3)	0.061 (3)	0.055 (3)	−0.023 (2)	0.007 (2)	−0.006 (2)
C38	0.092 (4)	0.106 (4)	0.069 (3)	−0.041 (3)	0.008 (3)	−0.012 (3)
C39	0.108 (5)	0.130 (6)	0.054 (3)	−0.035 (4)	0.015 (3)	−0.002 (3)
C40	0.089 (4)	0.076 (4)	0.094 (4)	−0.023 (3)	0.020 (3)	0.013 (3)
C41	0.106 (4)	0.088 (4)	0.091 (4)	−0.053 (3)	0.027 (3)	0.001 (3)
C42	0.085 (3)	0.087 (4)	0.072 (3)	−0.042 (3)	0.025 (3)	−0.019 (3)
N1	0.063 (2)	0.056 (2)	0.082 (2)	−0.0328 (18)	0.0229 (18)	−0.0271 (18)
N2	0.063 (2)	0.049 (2)	0.089 (2)	−0.0322 (18)	0.0261 (18)	−0.0242 (17)
N3	0.062 (2)	0.0433 (18)	0.072 (2)	−0.0322 (16)	0.0236 (16)	−0.0238 (15)
N4	0.0623 (19)	0.0468 (19)	0.065 (2)	−0.0313 (17)	0.0236 (16)	−0.0196 (15)
N5	0.056 (2)	0.048 (2)	0.101 (3)	−0.0282 (18)	0.0176 (19)	−0.0173 (19)
N6	0.058 (2)	0.0465 (19)	0.104 (3)	−0.0307 (18)	0.0243 (19)	−0.0248 (18)
N7	0.0534 (19)	0.0418 (18)	0.082 (2)	−0.0264 (16)	0.0183 (16)	−0.0238 (16)
N8	0.065 (2)	0.055 (2)	0.076 (2)	−0.0396 (18)	0.0288 (17)	−0.0303 (17)
O1	0.0621 (17)	0.0437 (17)	0.091 (2)	−0.0305 (15)	0.0205 (15)	−0.0235 (14)
O2	0.0660 (17)	0.0498 (17)	0.0777 (18)	−0.0317 (14)	0.0192 (14)	−0.0115 (14)
O4	0.0617 (17)	0.0434 (17)	0.096 (2)	−0.0289 (14)	0.0228 (15)	−0.0224 (15)
O5	0.117 (3)	0.094 (2)	0.078 (2)	−0.076 (2)	0.0206 (19)	−0.0272 (18)
O3	0.075 (2)	0.089 (3)	0.164 (4)	−0.037 (2)	0.050 (2)	0.000 (3)
O6	0.0564 (18)	0.097 (3)	0.120 (3)	−0.0218 (18)	0.0247 (18)	−0.054 (2)
S1	0.0587 (6)	0.0450 (6)	0.0840 (7)	−0.0289 (5)	0.0226 (5)	−0.0203 (5)
S2	0.0546 (6)	0.0436 (5)	0.0817 (7)	−0.0264 (5)	0.0176 (5)	−0.0201 (5)

### Geometric parameters (Å, °)

**Table:**

C1—O3	1.423 (7)	C22—H22C	0.9600
C1—H1A	0.9600	C23—O6	1.362 (5)
C1—H1B	0.9600	C23—C28	1.369 (7)
C1—H1C	0.9600	C23—C24	1.387 (7)
C2—C3	1.371 (7)	C24—C25	1.353 (6)
C2—O3	1.375 (5)	C24—H24	0.9300
C2—C7	1.380 (7)	C25—C26	1.384 (6)
C3—C4	1.376 (6)	C25—H25	0.9300
C3—H3	0.9300	C26—C27	1.353 (6)
C4—C5	1.382 (6)	C26—C29	1.471 (6)
C4—H4	0.9300	C27—C28	1.382 (6)
C5—C6	1.385 (6)	C27—H27	0.9300
C5—C8	1.470 (5)	C28—H28	0.9300
C6—C7	1.392 (6)	C29—N5	1.305 (5)
C6—H6	0.9300	C29—S2	1.732 (4)
C7—H7	0.9300	C30—N6	1.284 (5)
C8—N1	1.307 (5)	C30—N7	1.384 (5)
C8—S1	1.734 (4)	C30—S2	1.724 (4)
C9—N2	1.302 (5)	C31—O4	1.228 (4)
C9—N3	1.378 (5)	C31—N7	1.353 (5)
C9—S1	1.715 (4)	C31—C32	1.514 (5)
C10—O1	1.198 (4)	C32—N8	1.437 (5)
C10—N3	1.378 (5)	C32—C33	1.516 (7)
C10—C11	1.507 (5)	C32—H32	0.9800
C11—N4	1.445 (5)	C33—H33A	0.9600
C11—C12	1.511 (7)	C33—H33B	0.9600
C11—H11	0.9800	C33—H33C	0.9600
C12—H12A	0.9600	C34—O5	1.225 (5)
C12—H12B	0.9600	C34—N8	1.337 (5)
C12—H12C	0.9600	C34—C35	1.481 (6)
C13—O2	1.227 (4)	C35—C36	1.303 (6)
C13—N4	1.349 (5)	C35—H35	0.9300
C13—C14	1.478 (5)	C36—C37	1.450 (6)
C14—C15	1.330 (5)	C36—H36	0.9300
C14—H14	0.9300	C37—C38	1.374 (7)
C15—C16	1.452 (6)	C37—C42	1.389 (7)
C15—H15	0.9300	C38—C39	1.404 (8)
C16—C17	1.383 (7)	C38—H38	0.9300
C16—C21	1.390 (6)	C39—C40	1.377 (9)
C17—C18	1.364 (7)	C39—H39	0.9300
C17—H17	0.9300	C40—C41	1.342 (9)
C18—C19	1.345 (8)	C40—H40	0.9300
C18—H18	0.9300	C41—C42	1.373 (7)
C19—C20	1.350 (8)	C41—H41	0.9300
C19—H19	0.9300	C42—H42	0.9300
C20—C21	1.391 (7)	N1—N2	1.381 (5)
C20—H20	0.9300	N3—H3A	0.8600
C21—H21	0.9300	N4—H4A	0.8600
C22—O6	1.430 (8)	N5—N6	1.376 (5)
C22—H22A	0.9600	N7—H7A	0.8600
C22—H22B	0.9600	N8—H8	0.8600
			
C3—C2—O3	124.8 (4)	C27—C26—C29	122.2 (4)
C3—C2—C7	121.4 (4)	C25—C26—C29	120.2 (4)
O3—C2—C7	113.8 (4)	C26—C27—C28	122.4 (4)
C2—C3—C4	118.7 (4)	C26—C27—H27	118.8
C2—C3—H3	120.7	C28—C27—H27	118.8
C4—C3—H3	120.7	C23—C28—C27	118.9 (5)
C3—C4—C5	122.2 (4)	C23—C28—H28	120.6
C3—C4—H4	118.9	C27—C28—H28	120.6
C5—C4—H4	118.9	N5—C29—C26	122.5 (3)
C4—C5—C6	118.0 (4)	N5—C29—S2	113.4 (3)
C4—C5—C8	120.6 (4)	C26—C29—S2	124.0 (3)
C6—C5—C8	121.4 (4)	N6—C30—N7	120.2 (4)
C5—C6—C7	120.9 (4)	N6—C30—S2	115.0 (3)
C5—C6—H6	119.5	N7—C30—S2	124.7 (3)
C7—C6—H6	119.5	O4—C31—N7	122.2 (4)
C2—C7—C6	118.8 (4)	O4—C31—C32	123.6 (3)
C2—C7—H7	120.6	N7—C31—C32	114.1 (3)
C6—C7—H7	120.6	N8—C32—C31	112.0 (3)
N1—C8—C5	122.2 (4)	N8—C32—C33	110.8 (3)
N1—C8—S1	114.3 (3)	C31—C32—C33	108.2 (4)
C5—C8—S1	123.5 (3)	N8—C32—H32	108.6
N2—C9—N3	119.9 (3)	C31—C32—H32	108.6
N2—C9—S1	115.2 (3)	C33—C32—H32	108.6
N3—C9—S1	124.8 (3)	O5—C34—N8	122.2 (4)
O1—C10—N3	122.5 (3)	O5—C34—C35	122.8 (4)
O1—C10—C11	125.7 (4)	N8—C34—C35	115.0 (4)
N3—C10—C11	111.7 (3)	C36—C35—C34	122.4 (4)
N4—C11—C10	112.7 (3)	C36—C35—H35	118.8
N4—C11—C12	110.7 (4)	C34—C35—H35	118.8
C10—C11—C12	109.0 (3)	C35—C36—C37	127.8 (5)
N4—C11—H11	108.1	C35—C36—H36	116.1
C10—C11—H11	108.1	C37—C36—H36	116.1
C12—C11—H11	108.1	C38—C37—C42	117.4 (4)
O2—C13—N4	121.6 (3)	C38—C37—C36	120.0 (5)
O2—C13—C14	122.8 (3)	C42—C37—C36	122.5 (4)
N4—C13—C14	115.4 (3)	C37—C38—C39	120.1 (6)
C15—C14—C13	119.4 (4)	C37—C38—H38	119.9
C15—C14—H14	120.3	C39—C38—H38	119.9
C13—C14—H14	120.3	C40—C39—C38	120.7 (6)
C14—C15—C16	127.4 (4)	C40—C39—H39	119.7
C14—C15—H15	116.3	C38—C39—H39	119.7
C16—C15—H15	116.3	C41—C40—C39	119.0 (5)
C17—C16—C21	117.2 (4)	C41—C40—H40	120.5
C17—C16—C15	123.3 (4)	C39—C40—H40	120.5
C21—C16—C15	119.4 (4)	C40—C41—C42	121.1 (6)
C18—C17—C16	120.2 (5)	C40—C41—H41	119.4
C18—C17—H17	119.9	C42—C41—H41	119.4
C16—C17—H17	119.9	C41—C42—C37	121.7 (5)
C19—C18—C17	122.5 (6)	C41—C42—H42	119.2
C19—C18—H18	118.7	C37—C42—H42	119.2
C17—C18—H18	118.7	C8—N1—N2	112.0 (3)
C18—C19—C20	118.8 (5)	C9—N2—N1	112.1 (3)
C18—C19—H19	120.6	C9—N3—C10	124.9 (3)
C20—C19—H19	120.6	C9—N3—H3A	117.6
C19—C20—C21	120.6 (5)	C10—N3—H3A	117.6
C19—C20—H20	119.7	C13—N4—C11	120.7 (3)
C21—C20—H20	119.7	C13—N4—H4A	119.7
C16—C21—C20	120.5 (5)	C11—N4—H4A	119.7
C16—C21—H21	119.7	C29—N5—N6	112.9 (3)
C20—C21—H21	119.7	C30—N6—N5	112.2 (3)
O6—C23—C28	125.5 (5)	C31—N7—C30	126.0 (3)
O6—C23—C24	114.7 (4)	C31—N7—H7A	117.0
C28—C23—C24	119.7 (4)	C30—N7—H7A	117.0
C25—C24—C23	119.5 (4)	C34—N8—C32	122.6 (3)
C25—C24—H24	120.2	C34—N8—H8	118.7
C23—C24—H24	120.2	C32—N8—H8	118.7
C24—C25—C26	121.9 (4)	C2—O3—C1	117.5 (5)
C24—C25—H25	119.0	C23—O6—C22	117.9 (4)
C26—C25—H25	119.0	C9—S1—C8	86.3 (2)
C27—C26—C25	117.5 (4)	C30—S2—C29	86.49 (19)

### Hydrogen-bond geometry (Å, °)

**Table:**

*D*—H···*A*	*D*—H	H···*A*	*D*···*A*	*D*—H···*A*
N3—H3A···O4	0.86	2.10	2.960 (4)	177.
N4—H4A···N6^i^	0.86	2.17	3.016 (5)	168.
N7—H7A···O1^ii^	0.86	2.09	2.944 (4)	171.
N8—H8···N2	0.86	2.20	3.036 (5)	165.

Symmetry codes: (i) *x*, *y*−1, *z*; (ii) *x*, *y*+1, *z*.
